# Editorial: Multidisciplinary Approaches to Understanding Early Development of Spatial Skills: Advances in Linguistic, Behavioral, and Neuroimaging Studies

**DOI:** 10.3389/fpsyg.2021.666382

**Published:** 2021-04-09

**Authors:** Hui Li, Jin Sun, Xiao Zhang

**Affiliations:** ^1^Macquarie School of Education, Macquarie University, Sydney, NSW, Australia; ^2^Department of Early Childhood Education, The Education University of Hong Kong, Hong Kong, China; ^3^Faculty of Education, The University of Hong Kong, Hong Kong, China

**Keywords:** multidisciplinary approaches, early spatial skills, neuroimaging approach, linguistic approach, behavioral approach

Spatial cognition is a fundamental component of human cognition. Early spatial skills have longitudinal relations with later educational and occupational achievements in science, technology, engineering, and mathematics (STEM). Thus, it is critical to develop and deliver early interventions for young children to acquire spatial skills to lay a solid foundation for their future development. To achieve this goal, there is a need to understand how space is represented and presented by young minds and how young children acquire spatial language and skills using linguistic, behavioral, and neuroimaging approaches. This Special Collection represents an important endeavor to collect multidisciplinary studies on the early development of spatial skills and their implications for child development. As the guest editors, we strongly wish to advance the study on this topic by presenting this special collection.

## Early Spatial Skills: An Understudied Area

Spatial skills are a group of core cognitive abilities including spatial visualization (the ability to imagine and mentally transform spatial information), form perception (the ability to copy and distinguish shapes from other shapes, including symbols), and visual-spatial working memory (the ability to hold the locations of different objects, landmarks, etc.) (Rittle-Johnson et al., 2019). Young children regularly engage their spatial skills as they play blocks, puzzles, and videogames (Newcombe, 2010; Levine et al., 2012; Verdine et al., 2014; Jirout and Newcombe, 2015). Infants and toddlers also hear many spatial words when talking with their parents, and the frequency of hearing spatial words is a predictor of their development of spatial skills (Pruden et al., 2011). And mounting empirical evidence has suggested that spatial skills predict success in children's long-term development in the field of STEM (Wai et al., 2009; Newcombe, 2010; Uttal et al., 2013). Uttal and Cohen (2012) even regard spatial skills as a STEM “gateway.”

Despite the evidence, the importance of spatial skills is often overlooked in early childhood education. For instance, in the US, spatial skills have received minimal attention in the Pre-K and Kindergarten standards [National Council of Teachers of Mathematics, 2006; National Association for the Education of Young Children (NAEYC), 2014]. This public neglect of spatial development creates an additional barrier to children's early STEM learning. Therefore, there is growing advocacy for more attention to spatial skills early in education (Newcombe, 2010; Verdine et al., 2014). Further, there is much evidence to suggest that it is easy to foster early spatial learning as a core component of early childhood programs. Thus, research on the early development of spatial skills is urgently needed.

This Frontiers Research Topic aims at understanding how spatial skills develop in the early years (Age 0–8) from the perspectives of linguistics, behavioral science, and neuroscience. We have successfully collected nine cutting-edge studies to reflect the latest developments and advances in this rapidly emerging field. These articles were written by emerging and leading researchers from linguistics, psychology, education, neuroscience, and related fields. They have scrutinized spatial skills development in the early years using linguistic, behavioral, and neuroimaging approaches. This editorial will briefly review these studies and present some implications for future research.

## The Linguistic Approach to Studying Early Spatial Development

Spatial language lays the foundation for developing and learning spatial concepts. Studies on its acquisition using the linguistic approach can shed light on how the abstract concepts are acquired and constructed in the early years. Therefore, early spatial language acquisition and production studies are of great importance in understanding the complicated relationships between language and cognition in the early years (Majid et al., 2013). However, the existing studies on spatial terms have extensively adopted the experimental approach and only examined the acquisition of task-relevant spatial terms (Casasola et al., 2020), leaving the naturalistic production of spatial terms unexplored. This Research Topic thus deliberately collected three empirical studies on the natural language production of spatial terms.

Xu et al. article, “*The Use of a Novel Term Helps Preschoolers Learn the Concept of Angle: An Intervention Study with Chinese Preschool Children”*, opens this special issue with an early language intervention study aiming to foster preschoolers' conceptual understanding and linguistic presentation of “angle.” As an important concept in geometry, angle is widely used in daily communication and learning; but it is very abstract and difficult for young children to understand. This is because they have difficulties in differentiating “angle size” from “length of angle sides” due to limited word knowledge. This study adopted a quasi-experimental research design to investigate the effectiveness of two ways of separating angle from angle size in 3- to 6-year-old Chinese preschoolers. In this study, Xu et al. found that the experimental group improved significantly more than the other two groups. But, separating the words/phrases for angle and angle size might not help young children differentiate the two concepts, which share the same Chinese character/word “jiao” (angle). Some novel terms should be used to improve young children's learning. This finding indicates that language shapes or limits cognition in the early years, providing empirical evidence to the Whorf Hypothesis. This Hypothesis suggests that the structure of a language affects its speakers' world view or cognition, and thus people's perceptions are relative to their spoken language. Although still arguable and debatable, this Hypothesis proves to be true with preschoolers in this study.

In the second article, “*A Corpus-Based Comparison of the Pragmatic Use of Qian and Hou to Examine the Applicability of Space-Time Metaphor Hypothesis in Early Child Chinese*,” Tsung and Wu have examined the Universal Space-Time Mapping Hypothesis using natural language data elicited from a corpus. The Hypothesis suggests that temporal expression is based on spatial metaphor for all human beings and languages. Thus, this study explored its applicability in the Chinese language produced by 168 Mandarin-speaking preschoolers in a toy play context. To do so, they tested the use of the unique pair of Chinese words, qian (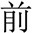/before/front) and hou (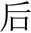/after/back), which could express either time (before/after) or space (front/back) in daily communication. They found a significant age difference and a critical period (before 4.5 years) in the pragmatic use. The pair was produced to express time (before/after) much earlier and more than space (front/back). Therefore, they concluded that time expression might not necessarily be based on the spatial metaphor, which challenged the Hypothesis.

The third article, “*Spatial Language of Young Children during Block Play in Kindergartens in Urban China*,” reports findings from a study on preschoolers' language use during block play. It is widely believed that spatial language can predict spatial skills and can be facilitated by peer interactions and goal-oriented building behaviors. In this study, Yang and Pan investigated the frequency, type, and level of children's spatial language and their associations with the level of block play by observing 228 young children. They found that young children used more words about spatial locations, deictic terms, dimensions, and shapes. But fewer words about spatial features or properties and spatial orientations or transformations were produced. In addition, most young children used gestures in conjunction with spatial deictic terms. Although very descriptive, this study has also provided empirical evidence to indicate the potential relationship between language and early spatial skills.

## The Behavioral Approach to Studying Early Spatial Development

Many traditional ways can facilitate early spatial development, such as paper folding, block building, fine arts, and painting. For instance, paper folding and block building are common activities in Chinese and Japanese kindergartens. Still, their potential contribution to early spatial skills has not received adequate attention in the literature. This special issue also collects four articles reporting behavioral studies on early spatial development.

The fourth paper, “*Exploring the Relationship between Parental Involvement, Paper Folding Skills, and Early Spatial Ability: A Mediation Model,”* presents an innovative study on traditional paper folding activities. In this study, Wu and Sun investigated whether and how paper folding skills could predict early spatial ability. To do so, they developed and validated a measure of paper-folding skills. They found a significant age effect in paper folding performance, and parental involvement could also contribute to the performance. Besides, paper folding skills could also contribute to early spatial development. Therefore, they established a mediation model of the relationship between parental involvement and spatial ability. This finding has revealed the educational values of paper folding and provided a reliable measure of paper folding skills, which have tremendous implications for early childhood education. This line of research deserves extending and further digging.

The fifth article, “*The development of spatial representation through teaching block-building in kindergartners,”* evaluates the effects of a block-building intervention on kindergarteners' spatial representation skills, using a quasi-experimental research design. In this study, Cai et al. delivered the well-planned block-building program to the experimental group, leaving those control group children to play with blocks freely. They found that the intervention significantly promoted Chinese kindergarteners' spatial representations. This finding has revealed the educational value of well-prepared block building activities and indicates a new research direction warranting further studies.

The sixth article, “*Spatial skills associated with block building complexity in pre-schoolers,”* also explores the educational values of block building activities in Chinese kindergartens. In this study, Zhang X. et al. investigated the relationships between six measures of spatial skills and block building complexity. They found that shape recognition, shape composition, and shape-recognition-by-gender interaction significantly predicted children's block building complexity. This finding has some implications for improving block building activities and enhancing early spatial complexity.

In the seventh article, “*The Effect of Finger Gnosis on Young Chinese Children's Addition Skills”*, Zhang L. et al. have explored the association between finger gnosis and arithmetic skills in Chinese children and the underlying mechanism. In the literature, finger gnosis has been found to facilitate children's spatial learning, which might help children develop a mature number line. First, they found that finger gnosis was significantly associated with addition performance. Second, they found that girls' finger gnosis was better than boys', and children with musical training outperformed those without the experience. Third, they found that the children with high finger gnosis performed better in number line estimation than those with low finger gnosis. Last, they found that the number line estimation fully mediated the relationship between finger gnosis and addition performance. These findings have jointly revealed the educational values of finger gnosis and provided practical implications for early childhood education.

The eighth article, “*Is Early Spatial Skills Training Effective? A Meta-Analysis,”* is a meta-analysis review conducted by Yang et al. They systematically analyzed 20 spatial intervention studies (2009–2020) with children aged 0–8 years and found the average effect size (Hedges's g) was 0.96 (SE = 0.10). In addition, they also analyzed the effects of several moderators such as the type of study design, sex, age, outcome category, research setting, and type of training. The results indicated that many training strategies or programs could significantly foster young children's spatial skills, such as hands-on exploration, visual prompts, and gestural, spatial training. This finding has provided implications for future research, policy-making, and practical improvement.

## The Neuroimaging Approach to Studying Early Spatial Development

The last article of this special issue, “*Neural Correlates of Mental Rotation in Preschoolers with High or Low Working Memory Capacity: An fNIRS Study,”* is an fNIRS study of the differentiated neural correlates of mental rotation (MR) in preschoolers with high and low working memory. Yang et al. tested 38 Chinese preschoolers with Working Memory Capacity (WMC), Mental Rotation, and Control tasks. They found no significant differences in MR task performance between the High- and Low-WMC groups. However, the two groups differed significantly in the activation of BA44 and BA9 during mental rotation. They concluded that BA9 and BA44 should be the neural correlates of mental rotation. This finding has provided neuroimaging evidence about the cognitive processing of mental rotation in preschoolers.

## Future Directions: Where Shall We Go?

This special edition calls for attention to the early development and facilitation of spatial skills, given its fundamental importance for future learning outcomes and significant literature gaps. The gaps include a lack of research on how children's spatial language works together with their spatial skills to facilitate their early cognitive development and other learning outcomes; how the facilitation of early spatial skills can be integrated into the early childhood education curriculum and be supported in children's everyday interactions with parents; and the neural mechanisms underlying early spatial learning and relevant impairment.

The existing studies have identified scattered relationships between spatial language and the mastery of particular spatial concepts and suggested that spatial language supports spatial skills proficiency. Such evidence is not able to provide a comprehensive picture of the relationship between spatial language and skills. With a group of core cognitive abilities including spatial visualization, form perception, and visual-spatial working memory, spatial skills deserve more systematic studies to further reveal how spatial language lays the foundation for developing different aspects of spatial concepts and skills.

Another consensus from the existing correlational and experimental studies is the neglected value of daily spatial activities, such as paper folding and block building, on early spatial development. It is worth paying more attention to children's daily spatial-related activities and integrating these activities in the systematic early childhood education curriculum and parenting education, given the popularization, playfulness, and potential educational benefits in such activities.

Last but not least, we still know very little about how the brain processes different forms of spatial information in the early years and whether such processes differ when children grow up. The fNIRS, a non-invasive neuroimaging technique, might provide potential to explore this area among young children due to its tolerance of children's head motion, physical movement, and relatively comfortable experiences compared to other invasive techniques. Future well-designed neuroscientific studies should extend this research line with more diverse samples and longitudinal designs to enrich the theory building of developmental cognitive neuroscience considering children's spatial skills.

## Author Contributions

All authors listed have made a substantial, direct and intellectual contribution to the work, and approved it for publication.

## Conflict of Interest

The authors declare that the research was conducted in the absence of any commercial or financial relationships that could be construed as a potential conflict of interest.
